# Hare's affairs: Lessons learnt from a noninvasive genetic monitoring for tracking mountain hare individuals

**DOI:** 10.1002/ece3.6676

**Published:** 2020-09-04

**Authors:** Laura Schenker, Kurt Bollmann, Maik Rehnus, Sabine Brodbeck, Felix Gugerli

**Affiliations:** ^1^ Swiss Federal Institute for Forest, Snow and Landscape Research WSL Birmensdorf Switzerland

**Keywords:** capture–mark–recapture, conservation, genotyping error rates, lagomorphs, nuclear microsatellites

## Abstract

Systematic monitoring of individuals and their abundance over time has become an important tool to provide information for conservation. For genetic monitoring studies, noninvasive sampling has emerged as a valuable approach, particularly so for elusive or rare animals. Here, we present the 5‐year results of an ongoing noninvasive genetic monitoring of mountain hares (*Lepus timidus*) in a protected area in the Swiss Alps. We used nuclear microsatellites and a sex marker to identify individuals and assign species to noninvasively collected feces samples. Through including a marker for sex identification, we were able to assess sex ratio changes and sex‐specific demographic parameters over time. Male abundance in the area showed high fluctuations and apparent survival for males was lower than for females. Generally, males and females showed only little temporary migration into and out of the study area. Additionally, using genotyped tissue samples from mountain hares, European hares (*Lepus europaeus*) and their hybrids, we were able to provide evidence for the first occurrence of a European hare in the study area at an elevation of 2,300 m a.s.l. in spring 2016. For future monitoring studies, we suggest to include complementary analysis methods to reliably infer species identities of the individuals analyzed and, thus, not only monitor mountain hare individual abundance, but also assess the potential threats given through competitive exclusion by and hybridization with the European hare.

## Graphical abstract

Based on noninvasive sampling for individual genotyping in a central alpine region, we confirm sex‐ and season‐specific survival rates of the elusive mountain hare, a species threatened by climate change. We provide the first evidence of a European hare being present in the study area, which is at the upper limit of its elevational range. Our findings highlight the vulnerability of mountain hare and the importance of combining species assignment and individual fingerprinting in genetic monitoring studies.

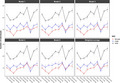

## 1 INTRODUCTION

Estimates of demographic parameters of populations provide important knowledge for the evaluation of conservation measures of a species (Allendorf, Luikart, & Aitken, 2013). Monitoring these parameters over time is essential to keep track of changes and to quantify threats to which species are particularly vulnerable (Lindenmayer & Likens, 2010).

Compared to traditional methods for monitoring wildlife populations (e.g., marking animals with unique identifiers), genetic monitoring may provide insights into more complex evolutionary and ecological processes (Schwartz, Luikart, & Waples, 2007), for example, patterns of gene flow (Carroll et al., 2018). Noninvasive genetic (NiG) sampling has become an established method for sample collection in genetic monitoring studies. This is particularly true for elusive, difficult‐to‐spot or rare animals (Beja‐Pereira, Oliveira, Alves, Schwartz, & Luikart, 2009; Waits & Paetkau, 2005), as individuals are genotyped based on remnants (e.g., feces or hair) collected in the field (Höss, Kohn, Pääbo, Knauer, & Schröder, 1992; Taberlet & Bouvet, 1992) and consequently do not need to be handled. NiG sampling may help avoiding stress or even death of individuals included in a monitoring. To apply such sampling in genetic monitoring studies, the classical capture–mark–recapture (CMR) framework (Luikart, Ryman, Tallmon, Schwartz, & Allendorf, 2010) has been modified accordingly (Lukacs & Burnham, 2005b). Since its introduction, NiG methods have been applied in numerous projects for the estimation of demographic parameters, for example, in wolf monitoring (*Canis lupus*; Dufresnes et al., 2019; Stenglein, Waits, Ausband, Zager, & Mack, 2010), the estimation of population abundance of grizzly (*Ursus arctos*) and black bears (*Ursus americanus*; Sawaya, Stetz, Clevenger, Gibeau, & Kalinowski, 2012), and for the estimation of contemporary dispersal and connectivity of capercaillie (*Tetrao urogallus*; Jacob, Debrunner, Gugerli, Schmid, & Bollmann, 2010; Kormann, Gugerli, Ray, Excoffer, & Bollmann, 2012; Rösner, Brandl, Segelbacher, Lorenc, & Müller, 2014). To date, nuclear microsatellites have been the most commonly used marker type for the application of NiG methods (Arandjelovic & Vigilant, 2018; Carroll et al., 2018), as they are highly variable due to high mutation rates (Waits, Luikart, & Taberlet, 2001). However, as animal remnants often contain DNA of poor quality and quantity, microsatellite genotyping of NiG samples may lead to high error rates, such as false alleles and allelic/genotypic dropout (Broquet & Petit, 2004; Taberlet, Waits, & Luikart, 1999; Waits & Leberg, 2000). High error rates in genotyping may lead to an over‐ or underestimation of census size (Creel & Rosenblatt, 2013; Lukacs & Burnham, 2005b; Waits & Leberg, 2000). The multi‐tube approach has been widely accepted to minimize these errors in NiG studies, but it also highlights the importance of study‐specific error estimations as well as species‐specific optimization of sampling strategies (Taberlet et al., 1996).

Here, we present a 5‐year NiG sampling dataset of the mountain hare (*Lepus timidus*) as study species, used to assess demographic parameters in a strictly protected area which allows their estimation under assumedly natural conditions (Rehnus, Marconi, Hackländer, & Filli, 2013). This enables us to draw conclusions about the conservation status and types of threat present in an exemplary alpine habitat. The mountain hare is classified as “Least Concern” by the International Union for the Conservation of Nature (Smith & Johnston, 2008), signifying sufficiently large global population sizes and no detectable or foreseeable significant decline in population abundance (IUCN, 2012). However, an increase in elevation and shift poleward has recently been predicted for mountain hare populations due to climate change (Leach, Kelly, Cameron, Montgomery, & Reid, 2015). For populations in the Alps, Rehnus, Bollmann, Schmatz, Hackländer, and Braunisch (2018) predicted a decline of suitable habitat and an increase in fragmentation due to changing climatic conditions. Besides having direct consequences for the mountain hare, climate change may also have indirect effects on mountain hare populations due to increasing competition with the European hare (*Lepus europaeus*). An extension of the range of the European hare into higher areas in the Alps can be expected from the positive effect of temperature increase (Thulin, 2003). As a consequence, the smaller mountain hare could be competitively excluded by the European hare (Acevedo, Jimenez‐Valverde, Melo‐Ferreira, Real, & Alves, 2012). Furthermore, hybridization between the two species could lead to introgression and threaten the genetic integrity of the mountain hare (Thulin, 2003; Thulin, Fang, & Averianov, 2006). Recent hybridization has been well documented in Scandinavia (e.g., Levänen, Thulin, Spong, & Pohjoismaki, 2018), whereas the current patterns of hybridization in the Alps are largely unknown (Beugin et al., 2017; Zachos, Slimen, Hacklander, Giacometti, & Suchentrunk, 2010). To understand the threats mountain hare populations are facing in the Alps, it is thus essential to monitor the occurrence of European hares and hybrids, in particular where species' elevational ranges overlap.

In this study, we investigate how NiG can be used to track population changes and to quantify threats for mountain hares by considering sex‐specific demographic parameters and tracking possible occurrence of European hares. In this way, we used the samples of an ongoing monitoring from the years 2014‒2018 and asked the following research questions: (a) Do apparent survival rates differ between sexes? As activity rates are sex‐specific during mating season and have been closely linked to survival (Murray, 2002), we expect to observe sex‐specific apparent survival rates. (b) Are temporary migration rates equal between sexes? Both sexes have been shown to display high site fidelity (Bisi et al., 2011); therefore, we expect to observe low temporary migration rates for both sexes. (c) Are European hares already present in the study area? Through genotyping tissue samples of known European and mountain hares and comparing the thereby obtained individual genotypes to individual genotypes obtained for the monitoring, we expected to be able to assign species identities (Beugin et al., 2017).

## 2 MATERIAL AND METHODS

### 2.1 Study area

**FIGURE 1:**
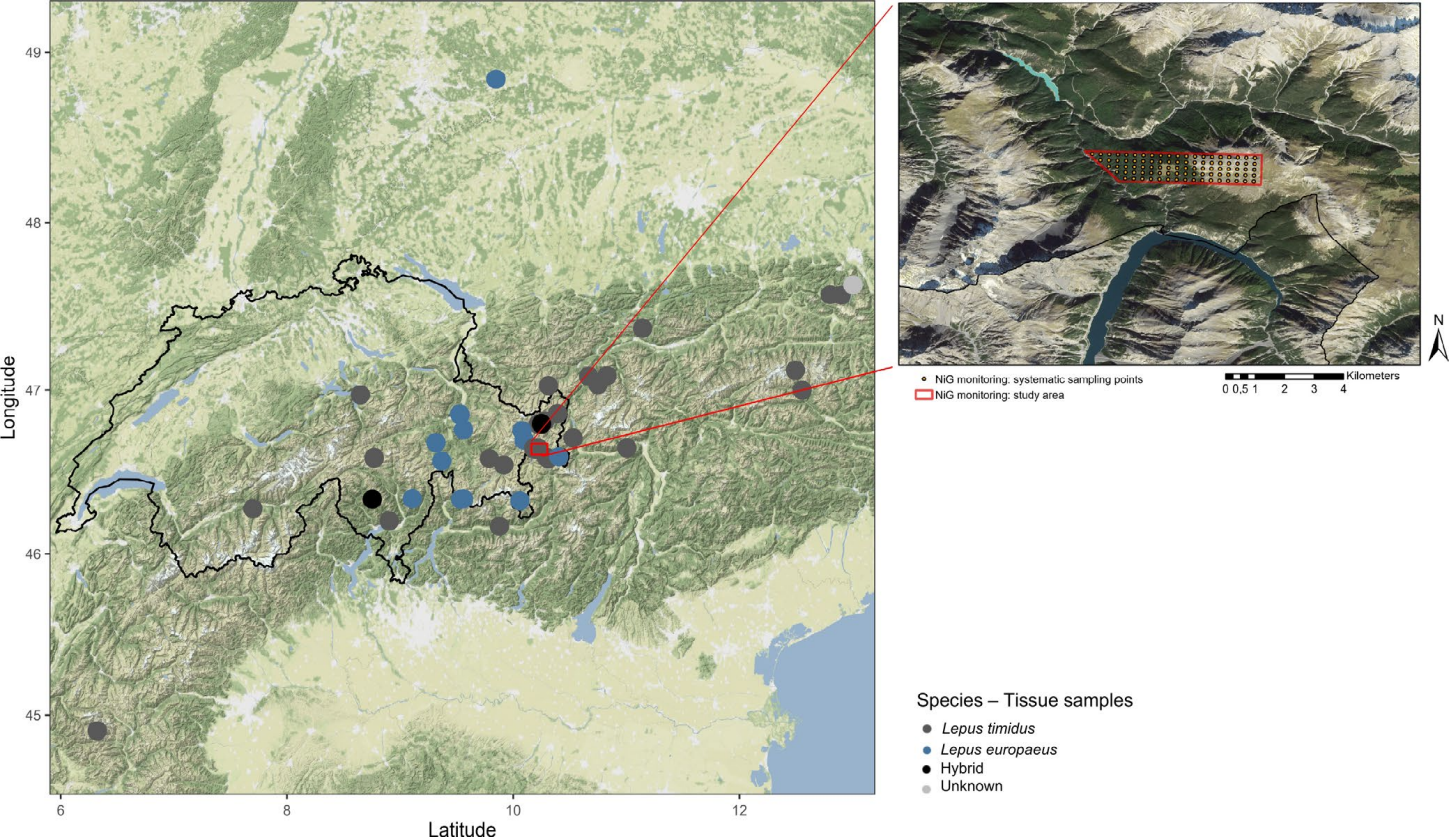
Origins of tissue samples (left, dots) and location and extent of the study area (right, red) in the Swiss National Park, with systematic sampling points (right, orange circles). On the left, origins of European hare (*Lepus europaeus*, Le) samples are displayed in blue, mountain hare (*Lepus timidus*, Lt) samples in dark gray, hybrids (Hb) in black, and samples of unknown species identity in light gray

Our study area is located in the Swiss National Park (46°39′N, 10°11′E), spanning an area of 3.5 km^2^; selected both to study mountain hare under natural conditions and to provide accessibility for sampling in rugged, hazardous alpine terrain in late winter (Figure 1, Rehnus & Bollmann, 2016). The Swiss National Park (IUCN Category 1a; IUCN, 2019) is inaccessible for the public in winter, but a popular area for recreational activities in summer, whereby visitors are restricted to use marked paths and areas.

### 2.2 Sample collection

We collected fresh fecal pellets during the mating period (end of March until first half of April) to detect individuals that potentially contributed to the next reproductive cycle (Luikart et al., 2010; Thulin, 2003) and during the postreproductive period (October) for recording offspring born in the summer of the same year in addition to adults (Pehrson & Lindlöf, 1984). Only fresh fecal pellets, which were identified based on surface characteristics, were collected, as Rehnus and Bollmann (2016) found significantly lower amplification success rates for pellets older than 5 days. Samples were collected both systematically and opportunistically, following the methods of Rehnus and Bollmann (2016). Systematic samples were collected at 91 sampling points on a 200 m square grid (Figure 1). Opportunistic samples were collected whenever fresh fecal pellets were found between systematic sampling points. To ensure that only fresh fecal pellets were collected, all present fecal pellets were cleared from every systematic sampling plot during a first survey and any fecal pellets detected during a subsequent survey (3–5 days later) were collected. It was assumed that feces with different shades of surface color found at the same location originated from different individuals or different dates, as previous studies have shown a considerable overlap in home ranges of mountain hare (Bisi et al., 2011; Rehnus & Bollmann, 2020). In these cases, we collected one feces per color type. In cases where two feces from the same color type were available, a backup fecal pellet was collected from each feces location to have a potential replicate for samples with low DNA quality (see *DNA extraction and PCR amplification*). All cases for which both the replicate and original sample were genotyped showed identical genotypes. Both systematic and opportunistic sampling were conducted within a short time span (maximally 11 days) in each sampling period, during which the population could be assumed as closed (Rehnus & Bollmann, 2016). Samples were collected and stored in separate plastic tubes without touching by hand to minimize DNA contamination (Sloan, Sunnucks, Alpers, Behregaray, & Taylor, 2000). After collection in the field, samples were frozen and stored until analysis in the laboratory.

For the possible identification of European hares, 90 tissue samples from known European and mountain hares and their hybrids were collected by hunters, game keepers, taxidermists, and other researchers at different locations across Europe (Figure 1) and frozen at −20°C until further analysis. Samples included different types of tissue, such as parts of the ear, paws, muscle, and bones. The species of each sample was determined by its collector based on morphological characteristics and classified—if possible—as either mountain hare (N_Lt_ = 51), European hare (N_Le_ = 36), hybrid of both hare species (N_Hb_ = 2), or else was classified as unknown species identity (N_NA_ = 1).

### 2.3 DNA extraction and PCR amplification

The following ten nuclear microsatellite loci were genotyped for the analysis of NiG samples (*N* = 1,637) and tissue samples (*N* = 90): Lsa1, Lsa2, Lsa3 (Kryger, 2002), Sat2, Sat5, Sat8, Sat12 (Mougel, Mounolou, & Monnerot, 1997), Sol30, Sol8 (Rico et al., 1994), and Sol33 (Surrige, Bell, Rico, & Hewitt, 1997). In addition, one marker was used to determine the sex (SRY; Wallner, Huber, & Achmann, 2001).

#### 2.3.1 Noninvasive genetic samples

DNA from fecal pellets collected in 2014 and 2015 was extracted after every sampling period following the protocol described by Rehnus and Bollmann (2016). DNA extraction from fecal pellets collected in 2016–2018 was performed with reagents from a customized sbeadex livestock kit (LGC Genomics) on a King Fisher Flex (Thermo Fisher Scientific). DNA samples were amplified as described in Rehnus and Bollmann (2016) in two multiplex PCRs with three independent replicates, following a modified multi‐tube approach (Taberlet et al., 1997, 1999). For amplification of samples from 2016 to 2018, concentrations of primers were lowered to 0.2–0.3 µM. If initial PCR results were negative, the backup fecal sample was used as replacement. Fragment length analysis of samples from 2016 to 2018 was performed on an ABI3130 genetic analyzer using GeneScan LIZ 500 Size Standard (Thermo Fisher Scientific), and electropherograms were visually analyzed using genemapper v5.0 (Thermo Fisher Scientific) after each sampling session.

#### 2.3.2 Tissue samples

DNA extraction for tissue samples was done using the DNeasy Blood & Tissue Kit (Qiagen) following the protocol for purification of total DNA from animal tissues (Spin‐Column Protocol, Qiagen). Pieces of 25 mg consisting of muscle, tendon, bone, or some hair were cut off from the samples and stored in 2 ml tubes at −20°C until further analysis. DNA concentration was assessed using quantus (QuantiFluor ONE System, Promega). The quality of the DNA samples was estimated with nanodrop 2000 (Thermo Fisher Scientific), based on the 260/280 nm ratio and concentration of the DNA, and quantitatively by the spectrum observed. Additionally, DNA quality was checked for all samples through electrophoresis using EZ‐Vision Blue Light Dye (Amresco, LLC) on a 1% agarose gel.

Based on the concentrations assessed using quantus (Promega), DNA samples were diluted to 2.5 ng/μl. Genotyping of tissue samples followed the protocol described above for NiG samples.

### 2.4 Genotyping

For tissue samples, PCRs not showing clear peaks in genemapper were repeated. For NiG samples for which multiple PCRs were run, consensus genotypes were created following the rules described in Table 1. The sex of tissue and NiG samples was assigned as male, if one or more samples amplified at the SRY locus, and as female, if no sample amplified.

**TABLE 1:** Examples for replicated genotyping results and their accepted consensus genotypes, applied for mountain (*Lepus timidus*) and European hare (*Lepus europaeus*) whenever replicates were available

Original replicate alleles						Consensus genotype	
Allele 1	Allele 2	Allele 1.1	Allele 2.1	Allele 1.2	Allele 2.2	Allele 1	Allele 2
A	A	A	A	A	A	A	A
A	B	A	B	A	B	A	B
A	B	A	B	A	A	A	B
A	B	A	B	NA	NA	A	B

Note

All replicate combinations given in the table were accepted, and combinations not listed were not accepted. NA values indicate allele dropout.

The three loci Sat2, Sat12, and Lsa2 could not be reliably scored as biallelic markers. Thus, they were not included in the automized multilocus genotype analysis. However, loci Sat2 and Sat12, but not Lsa2, showed consistent patterns of multiple allele peaks and stutter peaks among replicates and were thus scored qualitatively for NiG samples and used to distinguish among replicate genotypes with mismatching loci (see below). Multilocus consensus genotypes were accepted if at least six of the seven remaining loci could be scored successfully.

The raw genotype table output of the remaining seven loci was analyzed to find consensus genotypes for each replicated sample. Using a custom script in R v3.5.1 (R Core Team, 2018), we considered each replicate for which a genotype could be scored (positive PCR; Broquet & Petit, 2004) and applied the predefined conditions (Table 1): (a) Consensus homozygote genotypes were accepted if all three replicates were consistent, and (b) consensus heterozygote genotypes were accepted if at least two replicates were consistent and no more than two alleles were found across all three replicates.

Even though we applied stringent conditions, multilocus genotypes cannot be considered error free (Broquet, Menard, & Petit, 2007). Here, we quantified error rates as (a) missing replicate genotypes and (b) false homozygote replicates in consensus heterozygote genotypes. The rate of missing replicates was calculated in R as the proportion of missing replicate genotypes per sample in the raw genotype data. The rate of false homozygotes was estimated as the relative number of false homozygote replicates in consensus heterozygote genotypes, based only on sample/locus combinations for which a consensus genotype could be successfully scored as heterozygote.

Further types of quantified error included errors in sex determination. Sex determination using SRY is based on the amplification of the marker on the Y chromosome (Wallner et al., 2001). However, the nonamplification could either indicate a female individual or a dropout of the locus, and thus, certain errors are expected especially for female individuals.

### 2.5 Statistical analyses

#### 2.5.1 Genotype data

For both types of errors (false homozygote replicates and missing replicate genotypes), means for each season, year, and marker were calculated and compared using the package agricolae in R, which applies a pairwise comparison of group means including standard errors (*SE*) based on Tukey's honestly significant difference (Mendiburu, 2019). For the proportion of false homozygotes, we additionally assessed the amount of missing data in relation to the sex of the individual/genotype. Because the assessment of missing data was estimated based on raw genotype data and the sex could not be reliably determined for samples with large amounts of missing data, the amount of missing data could not be checked for differences between sexes.

Unique multilocus genotypes and unique genotype groups (individuals) in the NiG data set were identified based on successfully scored multilocus consensus genotypes using the allelematch package in R, which considers genotyping errors and missing data during the assignment of individuals (Galpern, Manseau, Hettinga, Smith, & Wilson, 2012). Subsequently, genotypes showing mismatches, female genotypes in male genotype groups, and multi‐match samples were double‐checked using the two previously excluded loci (Sat2, Sat12). Checking the additional loci was done qualitatively through comparing peak patterns in genemapper. Samples showing identical peak patterns (fragment lengths, patterns of stutter peaks) in genemapper were considered to originate from the same individual. Some examples of such peak patterns are given in Appendix S1. Samples were considered to belong to the same unique group of multilocus genotypes if they did not show more than two mismatched alleles including the additional loci. Females showing an exact match to male genotype groups were reclassified as males and marked as false females.

#### 2.5.2 NiG monitoring

The calculation of population genetic statistics and testing for Hardy–Weinberg equilibrium (Hardy, 1908; Weinberg, 1908) for the NiG genotype data was done in cervus v3.0.7 (Kalinowski, Taper, & Marshall, 2007). We calculated observed and expected heterozygosity, polymorphic information content (PIC), null allele frequency estimates, and the probability of identity under the assumption that individuals are unrelated (P_ID_) as well as under the assumption of siblings being present in the data (P_IDsib_). Loci for which potential null alleles were estimated to be present were still included in the analysis, as no true proof of null alleles can be given and estimates of null allele frequencies in cervus are based on homozygote frequencies (Kalinowski et al., 2007).

CMR methods seek to estimate animal abundance via capture of individuals (e.g., collection of genetic material) and marking (e.g., genetic identification) for future identifications (Williams, Nichols, & Conroy, 2002). The robust design (Pollock, 1982) distinguishes between primary and secondary sampling occasions, whereby primary sampling occasions (PSOs) are separated by enough time for population change to happen, and consist of one or more secondary sampling occasions (SSO), during which the population can be assumed as closed (Kendall, Nichols, & Hines, 1997). We applied the robust design model to estimate individual abundance, accounting for misidentifications in the data, under the assumption that genotyping errors could be present (Lukacs & Burnham, 2005a; Otis, Burnham, White, & Anderson, 1978), as implemented in mark v9.0 (White & Burnham, 1999). Each sampling day was considered as SSO and each sampling session (e.g., spring 2014) was considered as PSO.

Using individual detection histories based on (a) both systematically and opportunistically collected samples and (b) only systematically collected samples, we defined a set of plausible models, using sex as grouping variable. Thereby, we applied different assumptions for capture (p) and recapture (c) probabilities, the rate of misidentification (α), the assumed number of undetected individuals (f0), temporary emigration (γ′) and immigration (γ″), and apparent survival rates (φ). γ′,γ″, and φ are estimated for times between PSO (open population model), whereas p and c are estimated for each SSO (closed population model) and α and f0 are estimated for each PSO. Models in mark were set up to test for temporal and seasonal variation of the estimated parameters and for differences between the grouping variables. The models were ranked using Akaike's Information Criterion with correction for small sample sizes (AICc; Burnham & Anderson, 2002). After choosing the most informative model, the parameters were estimated across the most informative models as weighted means according to their support, as given by AICc weight, including weighted means for standard error (*SE*) to account for uncertainty in model selection. As derived parameter, mark estimates a census size estimate ($\hat{N}$) per PSO. All rates calculated were visualized using the R package ggplot2 (Wickham, 2016).

#### 2.5.3 Species assignment

Species assignment of individuals detected in the Swiss National Park based on NiG sampling were assessed through including a tissue data set consisting of high‐quality DNA samples of predefined species identity. To do so, a first analysis was conducted using only the tissue data set to identify whether differences between species could be detected with the loci used in the monitoring study. Thereafter, the second analysis was done using a combined data set consisting of both the tissue genotypes and the individual genotypes detected in the Swiss National Park from NiG sampling. First, a principal component analysis (PCA) was conducted in the R environment on a genind object in adegenet (Jombart, 2008), which is based on the package ade4 (Dray & Dufour, 2007), and the results were visualized using factoextra (Kassambara & Mundt, 2017). Assignments made by the PCA were then analyzed to look for individuals from the NiG dataset with higher similarity to European than to mountain hares. Second, to get further confirmation for species affiliations, individual assignments were assessed using structure v2.3 (Pritchard, Stephens, & Donnelly, 2000). The admixture model was applied for both data inputs, using a burn‐in period of 100'000 and 1'000'000 Markov Chain Monte Carlo (MCMC) repetitions after burn‐in. Additionally, the correlated allele frequency model was used, which provides greater power to detect distinct but closely related populations (Porras‐Hurtado et al., 2013). Values for K were set to 1–5, using ten iterations per K. The value of K best explaining the data was assessed using structure harvester v0.6.94 (Earl & vonHoldt, 2012). After estimating the value for K with the highest likelihood, the results for the runs of the respective K were combined using the Full Search Algorithm in clumpp v1.1.2 (Jakobsson & Rosenberg, 2007) and plotted in R. Cluster assignments were then visually compared to groupings observed using PCA to find correspondences or discrepancies.

## 3 RESULTS

### 3.1 Genotyping and distribution of errors

**FIGURE 2:**
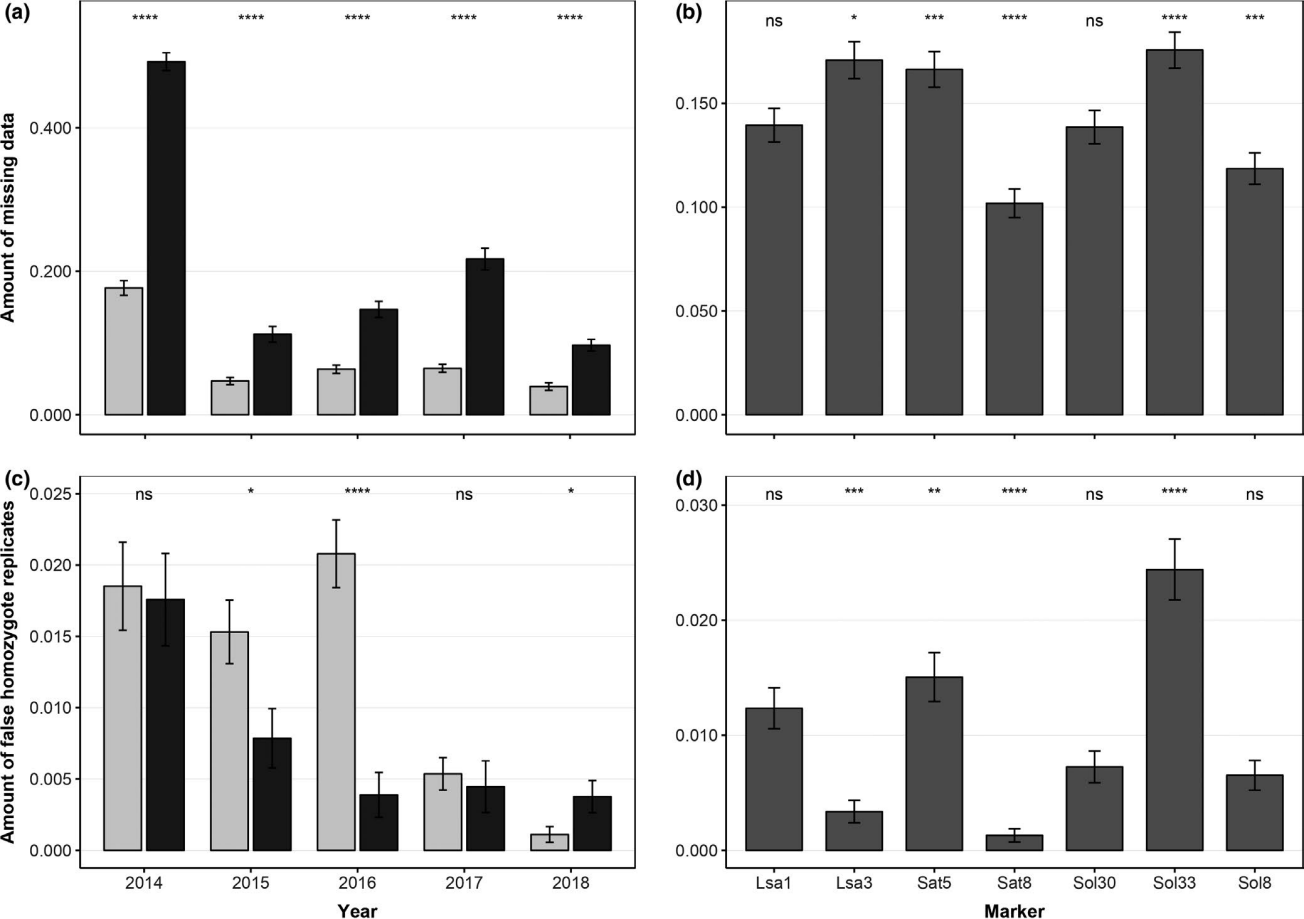
Error rate in the genotyping data obtained for mountain hares (*Lepus timidus*) based on noninvasive samples, as estimated by the proportional amount of missing replicates in replicate genotypes in each primary sampling period (a) and at each marker (b) and by the amount of false homozygote replicates and consensus heterozygote replicates (c, d). Significance codes refer to differences between sampling periods (a, c) or differences between markers (b, d). Spring sampling sessions are indicated in light gray, and fall sessions are colored dark gray. Values for each marker are given as seasonal averages, and error bars indicate standard errors

Over the five study years, 1,540 fecal samples were genotyped, while the remaining 97 samples failed due to negative initial PCR results. Of the former, 316 samples (20.5%) had to be removed due to too many missing loci in the consensus multilocus genotypes. The amount of missing data was significantly larger for samples collected in fall (P_NA_ = 0.24) than for samples collected in spring (P_NA_ = 0.07, *p* < .001), whereby the degree of the effect of season differed between years and was largest in the year 2014 (Figure 2a). The amount of missing data was significantly different between markers (*p* < .001), with Sat8 and Sol8 containing the lowest amounts of missing genotypes (Figure 2b). The number of false homozygotes was generally low, the highest amount was detected in spring 2016, when 2.1% of all consensus heterozygote replicates contained a false homozygote replicate (Figure 2c,d). The proportion of false homozygote replicates was approximately equal in spring (P_fh_ = 0.011) and fall (P_fh_ = 0.007). A significant difference between seasons was found only for the years 2015 (P_fh(spring)_ = 0.015, P_fh(fall)_ = 0.008, *p* < .05) and 2016 (P_fh(spring)_ = 0.021, P_fh(fall)_ = 0.004, *p* < .05, Figure 2c). In contrast, the proportion of false homozygotes was significantly larger in fall (P_fh_ = 0.004) than in spring (P_fh_ = 0.001, *p* < .05) in the year 2018 (Figure 2c). The number of false homozygotes in consensus heterozygote genotypes was significantly affected by loci Lsa3, Sat5, Sat8, and Sol33 (*p* < .001, Figure 2d). Sol33 contained the highest amount of false homozygote replicates and the highest amount of missing data (Figure 2b,d). The rate of false homozygotes and the rate of missing data consequently do not show the same patterns between loci or seasons.

On average, 4.4 alleles per locus were detected for individuals in the study area (Table 2). For loci Lsa3 and Sat5, the estimated null allele frequency indicates the possible presence of null alleles and an excess of homozygotes, respectively (Table 2). At locus Lsa3, allele 210 was by far the most common (P_210_ = 0.78) and most genotypes containing this allele were homozygous (87.5%).

**TABLE 2:** Overview of the population genetic parameters of mountain hare (*Lepus timidus*) in the study area in the Swiss Alps (2014–2018), calculated in cervus (Kalinowski et al., 2007): Number of alleles (A), number of individuals genotyped (N), observed (H_O_) and expected heterozygosity (H_E_), probability of identity (P_ID_), probability of identity under the assumption that siblings are present in the data (P_IDsib_), proportional amount of missing replicates (P_NA_) including standard errors of means (*SE*) across years, and the estimated null allele frequency (E(F_NULL_)), separately calculated for each locus and as mean across loci

Locus	A	N	H_O_	H_E_	P_ID_	P_IDsib_	P_NA_ ± *SE*	E(F_NULL_)
Lsa1	3	96	0.677	0.611	0.231	0.504	0.139 ± 0.008	−0.0567
Lsa3	5	93	0.140	0.371	0.416	0.669	0.171 ± 0.009	0.4645
Sat5	7	92	0.391	0.509	0.274	0.565	0.166 ± 0.009	0.1164
Sat8	2	96	0.146	0.136	0.757	0.872	0.102 ± 0.007	−0.0295
Sol30	6	96	0.396	0.426	0.365	0.629	0.139 ± 0.008	0.0375
Sol33	3	91	0.582	0.585	0.264	0.525	0.176 ± 0.009	−0.0010
Sol8	5	95	0.389	0.392	0.430	0.662	0.119 ± 0.008	−0.0006
Across	4.4	96	0.389	0.433	0.0008	0.036	0.144 ± 0.008	

The across‐locus P_IDsib_ = 0.036 (Table 2) was estimated based on the seven scored loci. However, for individual identification, loci Sat2 and Sat12 were qualitatively included and could thus not be used for the calculation of population genetic parameters. The inclusion of these loci would have decreased the calculated probabilities of identity.

### 3.2 Individual identification

**FIGURE 3:**
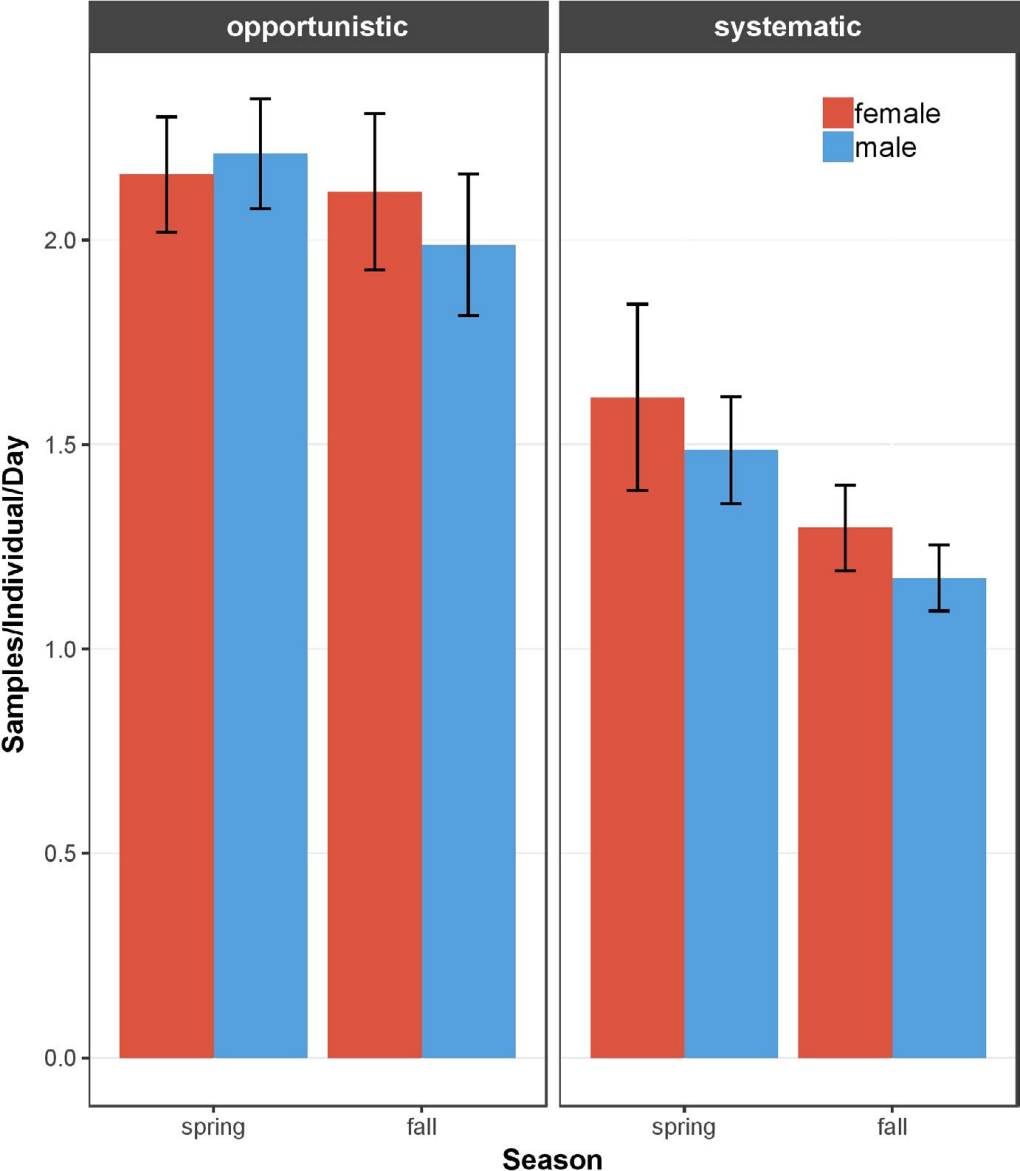
The mean number of samples collected per mountain hare (*Lepus timidus*) individual per day with each sampling method across all sampling periods (2014–2018) in the study area in the Swiss Alps. Opportunistic samples were collected whenever a fecal pellet of appropriate condition was detected between systematic sampling points. Values for females are given in red, values for males are in blue, and error bars indicate standard errors of means

From the 1,224 retained multilocus genotypes, 96 unique individuals were identified, of which 37 were classified as female and 59 as male. More samples originated from opportunistic sampling (N_opp_ = 992) than from systematic sampling (N_sys_ = 232). Across all sampling periods from both sampling methods, the mean number of samples collected per individual per day was 2.3 ± 1.8 (Mean ± *SE*), whereby the number of samples collected in spring (N_spring_ = 2.4 ± 0.1) was slightly higher than in fall (N_fall_ = 2.1 ± 0.1, *p* = .063). The number of samples collected per individual per day was lower for systematic sampling (N_sys_ = 1.4 ± 0.1) than for opportunistic sampling (N_opp_ = 2.1 ± 0.2, *p* < .001). For both sampling methods, no significant difference between seasons (p_(opp)_ = 0.391, p_(sys)_ = 0.107) or sexes (p_(opp)_ = 0.979, p_(sys)_ = 0.663) was detected (Figure 3). Consequently, both sampling methods revealed the same relative differences between sexes and season, but systematic sampling identified fewer individuals and individuals were identified based on fewer samples compared to opportunistic sampling.

### 3.3 Capture–Mark–Recapture analysis

#### 3.3.1 Model comparison

Of the 31 CMR models run, 21 models were unsupported, with model weights of less than 0.01. The ranking of the models did not show clear tendencies, as the four best models all showed a model likelihood of more than 0.25. Additionally, the model weight was mainly distributed among the 15 best fitting models, whereby the ten highest‐ranking models accounted for almost all the model weight (w_1_–w_10_ = 0.96, Table 3). For parameter estimation, we considered Models 1–5, as these models showed a model likelihood of more than 0.2, an AICc weight of more than 0.05, and a cumulative model weight of 0.85.

**TABLE 3:** Model comparison of the capture–mark–recapture models for mountain hare (*Lepus timidus*) obtained in mark (White & Burnham, 1999) for the ten highest‐ranking models (Models 1–10), including values for Akaike's Information Criterion with correction for small samples sizes (AICc), ΔAICc, AICc weight, model likelihood, and the number of parameters estimated

Model No.	Model	AICc	ΔAICc	AICc weight	Model likelihood	No. par.
1	φ(sex), γ′(.), γ″(.), p(season), c(season), α(season), f0(season)	1,779.2	0.00	0.29	1.00	12
2	φ(sex), γ′(.), γ″(.), p(sex, season), c(sex, season), α(sex, season), f0(sex)	1,779.4	0.11	0.27	0.95	18
3	φ(sex), γ′(.) = 1, γ″(.) = 0, p(sex, season), c(sex, season), α(sex, season), f0(sex)	1,780.8	1.60	0.13	0.45	16
4	φ(sex), γ′(.), γ″(.), p(sex, season), c(sex, season), α(sex, season), f0(season)	1,781.5	2.29	0.09	0.32	18
5	φ(sex), γ′(.), γ″(.), p(sex, season), c(sex, season), α(sex, season), f0(sex, season)	1,782.1	2.82	0.07	0.24	20
6	φ(sex), γ′(sex), γ″(sex), p(sex, season), c(sex, season), α(sex), f0(sex, season)	1,783.3	4.01	0.04	0.14	20
7	φ(sex), γ′(sex), γ″(sex), p(season), c(season), α(season), f0(season)	1,783.4	4.15	0.04	0.13	14
8	φ(sex), γ′(.), γ″(.), p(season), c(sex, season), α(sex, season), f0(sex, season)	1,783.8	4.58	0.03	0.10	18
9	φ(sex), γ′(.), γ″(.), p(sex), c(sex, season), α(sex, season), f0(sex, season)	1,783.8	5.97	0.01	0.05	18
10	φ(sex), γ′(.), γ″(.), p(sex, season), c(season), α(sex, season), f0(sex, season)	1,785.2	6.71	0.01	0.04	20

Note

All parameters were estimated either as constant (e.g., γ′(.) or γ′ (.) = 1), with variation by season (e.g., p(season)), with variation by sex (e.g., α(sex)), or with variation by both sex and season (e.g., f0(sex, season)).

The model with the best fit (Model 1) described unequal capture and recapture probabilities, variation for φ by sex, variation for α, f0, p, and c by season, and constant values for γ″ and γ′. However, the model with the second best fit (Model 2) estimated α, p, and c with variation by sex and season, φ and f0 with variation by sex and constant values for γ″ and γ′ (Table 3). Models considering equal capture and recapture probabilities generally showed a lower ranking than models with unequal probabilities. Additionally, models considering time dependence of variables showed lower rankings than models in which parameters were assumed to be constant through time. For example, none of the ten highest‐ranking models considered time dependence for survival. Time is considered per PSO for parameters estimated per SSO (p and c) and across PSO for parameters estimated per PSO (φ, γ″, γ′, α, f0).

#### 3.3.2 Parameter estimation

Models 1–5 described survival to be constant through time; thus, no seasonal or annual survival rates were estimated. Based on these models, females showed a higher apparent survival probability than males (Table 4).

**TABLE 4:** Parameter values as weighted means across capture–mark–recapture Models 1–5 for mountain hare (*Lepus timidus*), standard error and 95% confidence intervals (95% CI) as weighted mean across models (capture rate (p), recapture rate (c), rate of misidentification (α), number of missed individuals (f0), rate of temporary emigration (γ′) and immigration (γ″), and apparent survival rate (φ)) or as given by mark (White & Burnham, 1999) for the specific model (f0)

Parameter	Season	Sex	Mean	*SE*	95% CI	Type of mean
p	Fall	Female	0.25	0.04	0.19 – 0.32	Weighted mean (Model 2–5)
p		Male	0.17	0.02	0.13 – 0.22	
p	Spring	Female	0.26	0.03	0.19 – 0.33	
p		Male	0.26	0.03	0.20 – 0.33	
c	Fall	Female	0.22	0.04	0.15 – 0.31	
c		Male	0.21	0.04	0.15 – 0.29	
c	Spring	Female	0.34	0.04	0.27 – 0.41	
c		Male	0.44	0.03	0.38 – 0.51	
α	Fall	Female	0.81	0.10	0.56 – 0.93	
α		Male	0.77	0.11	0.50 – 0.91	
α	Spring	Female	0.95	0.05	0.70 – 0.99	
α		Male	0.77	0.06	0.63 – 0.87	
f0	Constant	Female	0.55	0.51	0.13 – 2.64	Model 2
f0		Male	2.01	0.88	0.89 – 4.59	
γ′	Constant	Constant	0.77	0.30	0.22 – 0.99	Weighted mean (Model 1–5)
γ″		Constant	0.05	0.04	0.01 – 0.21	
φ	Constant	Female	0.90	0.05	0.75 – 0.96	
φ		Male	0.76	0.05	0.64 – 0.85	

**FIGURE 4:**
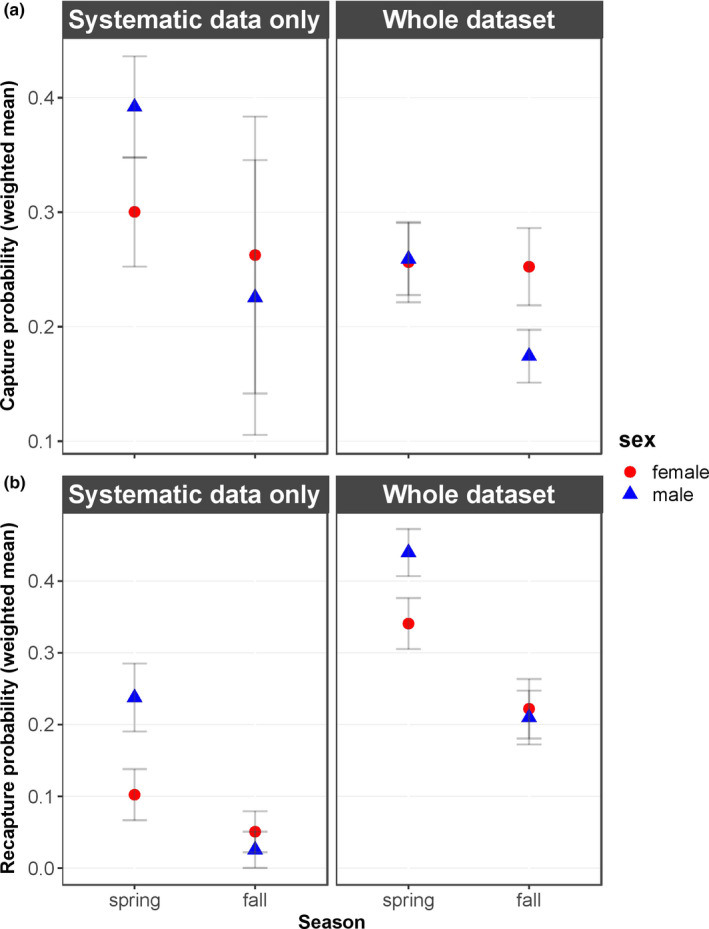
Overview of the weighted mean of capture (a) and recapture probabilities (b) calculated for the models using capture histories based on systematically collected samples and the whole dataset (systematic and opportunistic), given for male and female mountain hare (*Lepus timidus*) individuals. Error bars indicate weighted means of standard errors calculated by mark (White & Burnham, 1999)

In spring, recapture probabilities were higher than capture probabilities and both were higher than in fall for both sexes. For males, the difference between seasons was more pronounced than for females (Figure 4), as both capture and recapture probabilities were higher in spring than in fall (Figure 4, Table 4). Consequently, more male genotypes were observed in spring compared to fall for both newly identified and previously identified individuals. For females, capture probabilities were equal between seasons and recapture probabilities showed a less pronounced difference between seasons than for males (Figure 4, Table 4). Additionally, recapture probabilities were higher than capture probabilities in spring and equal to capture probabilities in fall (Table 4). Therefore, the probability to observe a new female was approximately equal in both seasons, but the probability to observe a new male was higher in spring than in fall.

The probability of temporary emigration between two subsequent PSOs (γ″) was estimated to be 0.05 ± 0.04, and the return rate of temporary emigrants (γ′) was estimated to be 0.33 ± 0.30 for both sexes (Table 4). Additionally, the no‐movement model was ranked relatively high (Model 3, Table 3). Consequently, temporary immigration and emigration (migration) into and out of the study area are assumed to occur at a similarly low rate for females and males.

In both seasons, the probability of correctly identifying an individual (α) was larger for females than males. Females showed a higher α in spring (0.95 ± 0.05) than in fall (0.81 ± 0.10), and α was the same for males in both seasons (0.77 ± 0.11). Model 1 estimated f0 to be variable only by season and estimated more individuals to be undetected in fall than in spring. Model 2 estimated f0 to be variable by sex and estimated more males to have remained undetected per primary sampling occasion than females (Table 4).

### 3.4 Systematic sampling

The models set up using capture histories based on only systematically collected data had relatively large standard errors for capture probabilities (Figure 4a), but showed similar patterns for recapture probabilities (Figure 4b). Capture and recapture probabilities were lower compared to the probabilities calculated by the models using capture histories based on both systematic and opportunistic samples (Figure 4). Thus, seasonal and sex‐specific patterns of recapture probabilities were confirmed using systematic sampling.

### 3.5 Individual abundance

**FIGURE 5:**
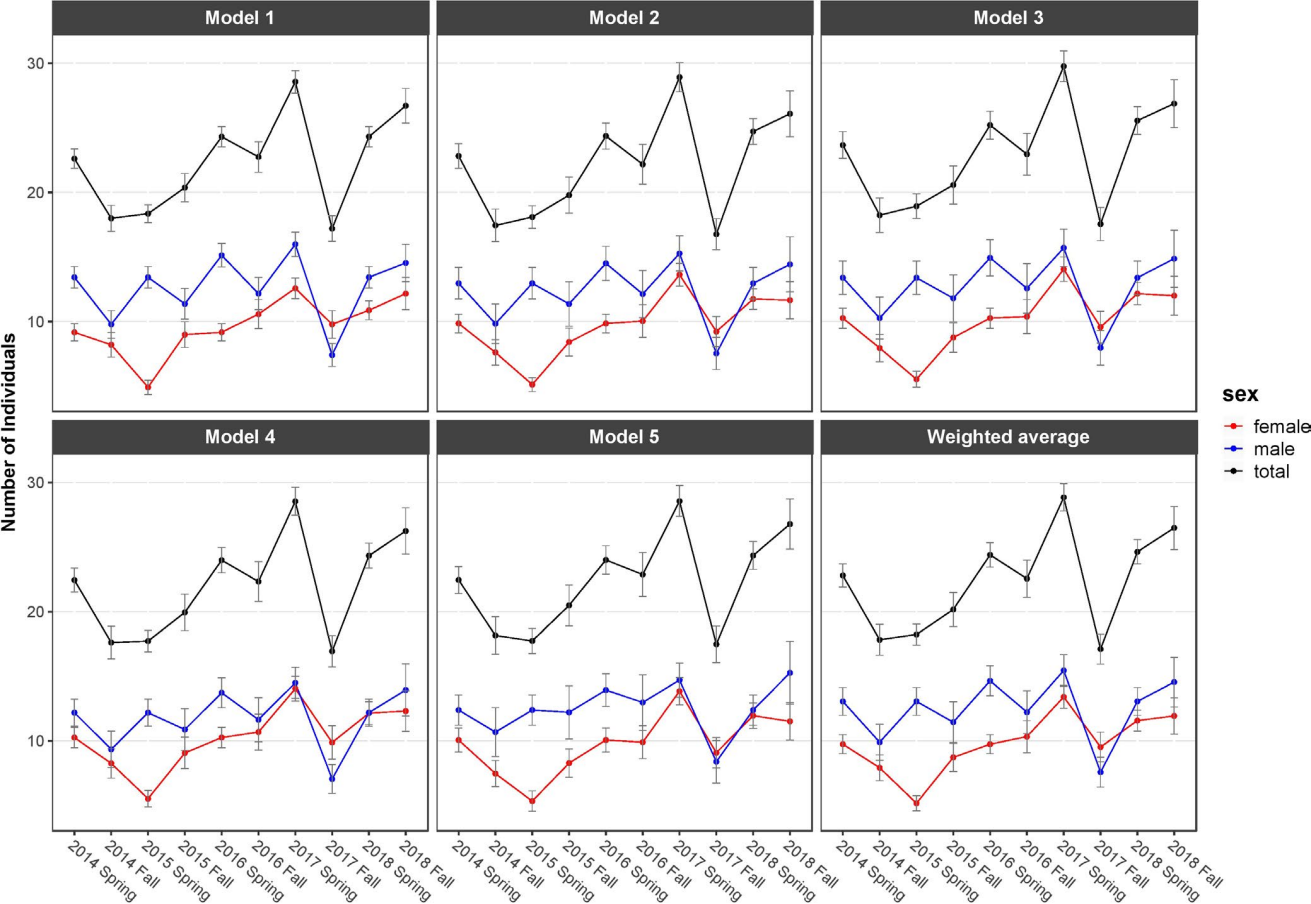
Individual abundance of mountain hare (*Lepus timidus*) in the study area in each primary sampling period, as given by each of the five best fitting models in mark (White & Burnham, 1999) and as their weighted average. Error bars indicate standard errors as estimated by mark for the model

Abundance in the study area was estimated to fluctuate between 30 and 15 individuals, whereby females showed a slightly positive trend and males showed larger fluctuations over time (Figure 5). Abundance was at a minimum in fall 2017 (${\hat{N}}_{2017F}$ = 17.1 ± 1.1, Mean ± *SE*), when fewer females (−28.9%) and fewer males (−50.9%) were present compared to spring 2017 (${\hat{N}}_{2017S}$ = 28.8 ± 1.1). However, the decrease in males from spring to fall 2017 was more pronounced than the decrease in females (Figure 5).

The male–female sex ratio was slightly larger in spring (1.53 ± 0.51) than in fall (1.15 ± 0.61). The sex ratio was largest in spring 2016 (2.5 ± 1.0) and lowest in fall 2016 (0.80 ± 0.44). Additionally, fall 2017 was the only sampling session for which more females than males were estimated to be present in the study area (Figure 5).

On average, a density of 6.4 hares per km^2^ was observed. The maximum density in the study area was 8.3 hares per km^2^ and occured in spring 2017. In fall 2017, the density was minimal with 4.9 hares per km^2^. One individual (LtNP02, female) was detected in every PSO from spring 2014 to fall 2018.

### 3.6 Species assignments

**FIGURE 6:**
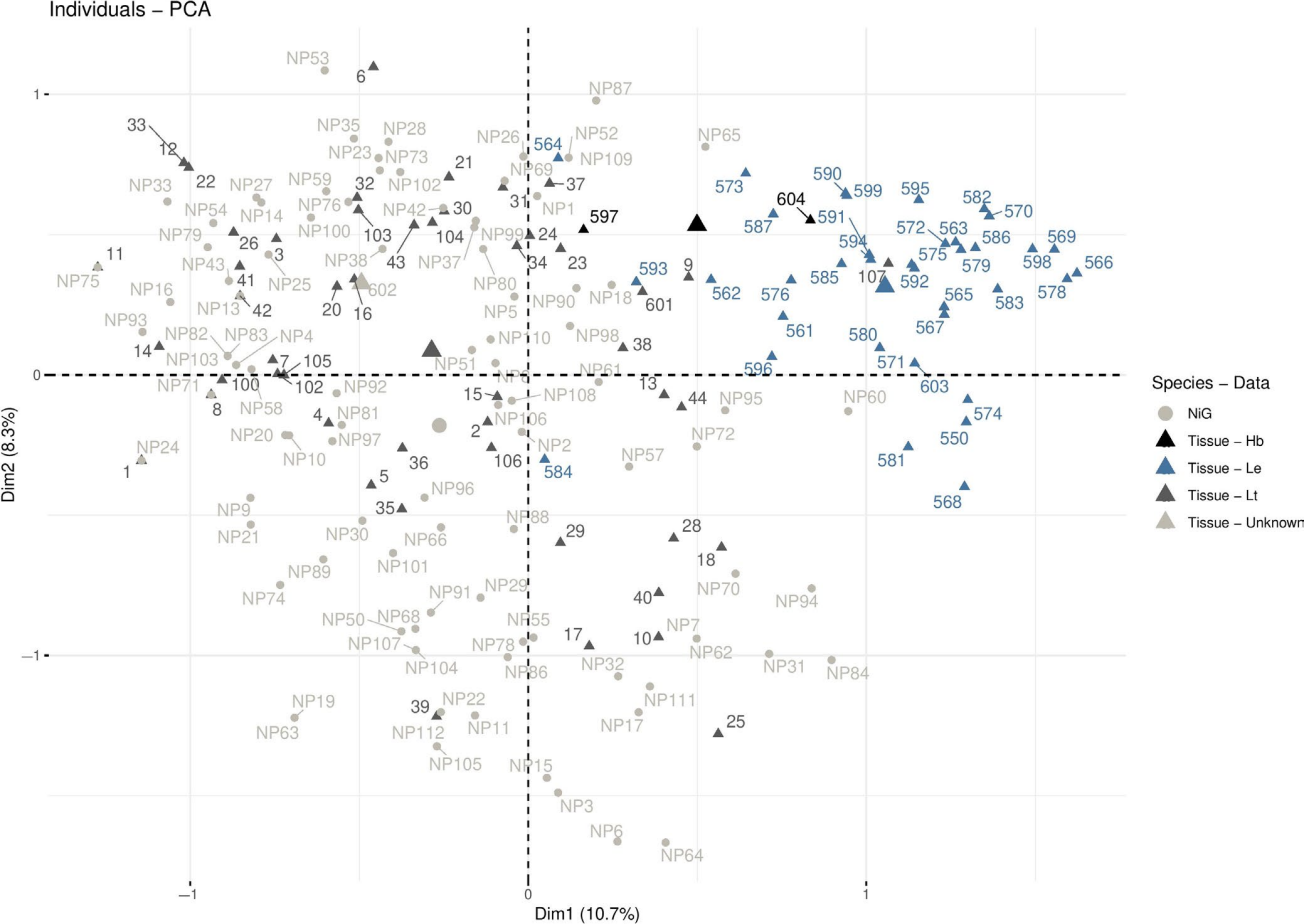
Results of the principal component analysis for genotypes of noninvasive genetic samples (light gray circles) and of tissue samples (triangles). Some samples morphologically assigned to *Lepus timidus* (Lt, dark gray) showed signs of being assigned to *L. europaeus* (Le, blue) and vice versa. One hybrid (Hb, black) sample was assigned to *L. europaeus,* and one hybrid sample was assigned to *L. timidus*. The sample of unknown origin (Unknown, light gray) was assigned to *L. timidus*. The large symbols represent the centroids of the defined groups

**FIGURE 7:**
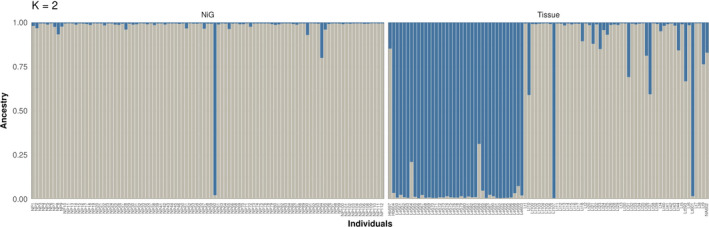
Results of the analysis run in structure (Pritchard et al., 2000): Cluster assignment probabilities are given in gray for *Lepus timidus* and in blue for *L. europaeus*. Individual genotypes identified based on noninvasive samples are named with the prefix “NP” and depicted on the left side. The morphological species assignments of tissue samples are given as “Le” for European hares, “Lt” for mountain hares, “Hb” for hybrids, and “NA” for unknown species identity

The PCA based on tissue sample genotypes with predefined species identities showed that most samples of the data set were genetically similar to each other at the loci assessed (Appendix S2). Nevertheless, the representation of the PCA depicted a lower left shift for most *L. timidus* samples and an upper right shift for most *L. europaeus* samples, also for the combined dataset consisting of NiG and tissue samples. Hereby, the two most informative axes accounted for 19.3% of the total variation (Figure 6). Some of the samples contained in the tissue dataset showed different species assignments to the ones defined by their collectors (e.g., Lt107, Figure 6). The presumed hybrid samples contained in the tissue dataset were shown to be either more similar to mountain hare (Hb597) or to European hare (Hb604). The combined dataset further showed two samples from the NiG monitoring dataset (LtNP60, LtNP95) to be more similar to European hare than mountain hare samples (Figure 6).

structure harvester (Earl & vonHoldt, 2012) revealed the most likely number of clusters for the combined and for only the tissue genotype data to be K = 2 (Figure 7), which is expected in a two‐species case. Generally, species assignments obtained for the tissue genotypes were mostly in accordance with the results obtained by the PCA. However, individual LtNP95 only showed slight signs of admixture, even though its position in the PCA plot was in‐between samples of the two hare species. Unexpectedly, individual Lt107 from the tissue dataset, morphologically identified as *L. timidus*, was clearly confirmed to belong to the *L. europaeus* cluster by both structure (P_LE_ = 0.995) and PCA (Figures 6 and 7). Further, the assignment of LtNP60 to the *L. europaeus* cluster was confirmed by structure at a high probability (P*_Le_* = 0.98). Notably, individual LtNP60 was genotyped based on ten samples collected in spring 2016 at a maximum elevation of 2,300 m a.s.l. in the study area.

## 4 DISCUSSION

In this study, we show the results of a multi‐year NiG monitoring on the elusive mountain hare. Through genotyping nuclear microsatellite loci and a sex marker based on DNA extracted from fecal pellets, we were able to show that male mountain hare individuals not only had lower apparent survival rates than females, but also higher fluctuations in activity rates throughout the year, presumably based on their mating behavior. We also found equally low temporary migration rates for males and females, which can be explained by the high site fidelity of both sexes. Further, we were able to show that a European hare had been present in the Alpine habitat at a maximal elevation of 2,300 m a.s.l., which marks the first observation of this species in the study area.

### 4.1 Density of individuals

We found that density of individuals in the Swiss National Park was on average nearly twice as large (6.4 hares per km^2^) as previously estimated (Rehnus & Bollmann, 2016). Rehnus and Bollmann (2016) used samples from one sampling session (spring 2014) and derived spatial capture–recapture estimates, which consider a buffer around the study area (Efford & Fewster, 2013). However, the density values previously estimated in this study area and in a close‐by location in Italy (5–11 hares per km^2^; Nodari, 2006) are also higher than those observed in a National Park in Austria (0.4–0.7 hares per km^2^; Slotta‐Bachmayr, 1998) and in Ticino, Switzerland (3.5 hares per km^2^; Gamboni et al., 2008). Besides each of these studies applying different methods and being conducted during different seasons, climatic conditions differ throughout the biogeographic regions of the Alps (Rehnus et al., 2018). Hence, comparisons of such density estimates should be taken with caution.

### 4.2 Fluctuations in census estimates

Seasonal fluctuations of the estimated census size were mostly due to fluctuating abundance of male individuals, as more individuals were present during the mating season than in the postreproductive period. Apparent survival was lower for males than females, male individuals mostly disappeared between spring and fall, and new male individuals appeared between fall and spring. Equally low amounts of temporary migration for both sexes confirmed the high site fidelity of mountain hares (Bisi et al., 2011). We assumed that this pattern can be explained by the range use of individuals, which often depends on the mating system (Ostfeld, 1990). The distribution of males is mainly determined by the availability of potential mates, whereas range use of females is mostly affected by the availability of food and shelter for nursing offspring (Bisi et al., 2011). Our spring sampling sessions coincided with the beginning of the mating season (April; Thulin & Flux, 2003), during which males are searching for mates. During this time, home ranges have been found to be enlarged compared to fall (September–November) for both sexes and to be larger for males than females (Sweden, Dahl & Willebrand, 2005). This was also found to be the case in the Alps, and as a consequence, home ranges also overlapped more strongly during this time, leading to more individuals being present (Bisi et al., 2011; Gamboni et al., 2008). We therefore conclude that males increase their home ranges during mating season, leading to more males being detected in the study area during this time of the year. Further, we conclude that the survival of males is lower than of females, leading to sex‐specific fluctuations in abundance.

### 4.3 Effects of sex and season on recapture rates

Our results show that individuals of both sexes had higher recapture probabilities during mating season than during the postreproductive period, whereby recapture probabilities were higher for males than females. From an ecological perspective, we explain higher recapture rates by higher activity rates. For example, hares are assumed to be more active during the mating season (Bisi et al., 2011), and male snowshoe hares (*L. americanus*) have also been found to increase their activity during this time of year (Murray, 2002). Higher activity of hares has been closely linked to lower survival probabilities via increased predation risk (Murray, 2002). Bisi et al. (2011) hypothesized that the reduction in home range sizes in fall, could be an antipredatory strategy, that is, to reduce predation risk while being midmolt.

From a methodical perspective, differences in genotyping success rates may cause differences in capture and recapture probabilities, as only successfully genotyped samples are recorded as capture occasions (Lukacs & Burnham, 2005a). We found genotyping success rates to be constant between sexes, but higher in spring than in fall. To control for a biased effect due to detectability and genotyping success caused by season‐specific sampling conditions, individual histories based on systematically detected samples were analyzed. Due to the sampling scheme, detectability is thought to only minimally influence the number of samples collected using systematic sampling. Based on these models, differences in capture and recapture probabilities between sexes and seasons were confirmed. This indicates that seasonal and sex‐specific variance is largely due to individual activities and reflects ecological conditions with higher activity rates and larger home ranges of individuals—especially of males—during mating season. This difference leads to higher individual abundance and a higher number of samples collected per individual, whereas a reduction in home range sizes in fall leads to lower recapture rates.

### 4.4 Monitoring European hare occurrence with implications for hybridization

One individual detected in our study area was identified as European hare. The individual (male, LtNP60) was found to be present in the park only during spring 2016 at a maximum elevation of 2,300 m a. s. l. Species assignment of this individual was confirmed using mtDNA sequencing (CoI; S. Brodbeck, unpublished data). mtDNA sequences differ between mountain and European hares and can thus be used for reliable species identification (Thulin, 2003; Thulin, Stone, Tegelstrom, & Walker, 2006).

With climate change, habitat of European hares is thought to experience an upward shift, which may cause European and mountain hare habitat to increase in overlap (Acevedo et al., 2012; Rehnus et al., 2018), intensifying competition between the two species and causing both competitive exclusion of the mountain hare and potential hybridization (Thulin, 2003). Here, we detected one individual of European hare at its upper end of the current elevational distribution range (Bauer, 2001; Bisi, Wauters, Preatoni, & Martinoli, 2015). While at least one further individual showed signs of admixture, we consider a better resolution of markers to be necessary to clearly detect admixture between both species. With our marker set, structure and PCA did not always show consistent results, but also morphologically based identification proved potentially inconclusive. To address this phenomenon of hybridization more reliably, genomic methods are thought to provide better resolution (Allendorf, Leary, Spruell, & Wenburg, 2001; Carroll et al., 2018). We recommend future work to develop a diagnostic panel of single‐nucleotide polymorphisms (SNPs), including mtDNA sequences for species diagnostic purposes (Thulin, Fang, et al., 2006), for integration into the standardized future monitoring to quickly assess species associations of the individuals detected.

### 4.5 Caveats and limitations

While the value of 0.036 for the probability of identity under the assumption of the individuals in the sample being related (Waits et al., 2001) is not particularly low, it still lies below the recommendation of the upper threshold given by Woods et al. (1999). The probability of identity (Waits & Leberg, 2000) is a proxy for the power of a set of markers and depends on the number of markers, the allele numbers and their frequency distributions, and the typing error rate of each marker (Wang, 2006). Even without error in the data, there is a probability that random individuals share a common genotype, which may lead to misidentifications of individuals (Mills, Citta, Lair, Schwartz, & Tallmon, 2000). However, the identification of unique genotypes was done with two additional loci than those included in the calculation of the P_IDsib_ value, for which electrophoresis peak patterns were consistent but did not allow biallelic scoring. Integrating this information in the calculation would lower the overall P_IDsib_ value (Wang, 2006). Including the two additional loci showed to be highly relevant. A few genotypes were identical at the applied seven loci, but showed distinctively different peak patterns at the additional loci (see Appendix S1 for examples). Therefore, interpreting banding patterns of two additional loci allowed us to resolve certain genotype groups into distinct individuals.

In our study, error rates differed between seasons and declined throughout the study years, but were constant between sexes. Throughout all years, we reduced errors by applying a modified multi‐tube approach based on Taberlet et al. (1996), performing three PCRs per sample. Further, we applied a strict rule in detecting homozygotes to ensure a minimum allelic dropout rate and increase the probability of consensus homozygous genotypes to be true homozygotes (Taberlet et al., 1996). The dropout rate across loci and years of approximately 20% is in accordance with observed dropout rates in other NiG studies (e.g., Ebert, Sandrini, Spielberger, Thiele, & Hohmann, 2012; Hansen, Ben‐David, & McDonald, 2008).While such restrictive genotype calling limited the overall genotyping success rate, it allowed us to keep the error rate at a reasonably low level.

Error reduction strategies and error quantifications were done consistently across years. We assume that error variation between seasons originates mostly from sampling conditions. In spring, samples were preserved by snow and low temperatures, whereas in fall, samples were less well preserved due to higher temperatures and sampling without snow. Further, Murphy, Waits, and Kendall (2003) observed that nutritional composition in bear feces causes variability in observed DNA qualities and quantities and therefore also in observed error rates. Seasonal differences in genotyping errors have also been observed for ungulates (Maudet, Luikart, Dubray, Von Hardenberg, & Taberlet, 2004). As food composition for mountain hares is different in fall than in spring (Rehnus et al., 2013), this aspect could be an additional factor causing the observed seasonal variability in error rates. We therefore conclude that genotypes of samples collected in fall, and consequently individual identifications, are less reliable than those of spring samples.

Some errors were further observed during the determination of sex. Even after removing samples with too many missing loci in consensus genotypes, some samples classified as females showed matches to groups of male genotypes and were consequently marked as false females (0.9%). Further support for this classification is given by the fact that the amount of missing genotypes was higher for false female samples than for all other samples (P_(NA, false females)_ = 0.06, P_(NA, females)_ = 0.01). A certain error rate in sex determination using SRY was also detected by Wallner et al. (2001). This finding highlights the importance of replicating noninvasively collected samples and of using an adequate number of samples for individual identifications.

## 5 CONCLUSIONS

In this study, we show how sex‐specific demographic parameters of an elusive and difficult‐to‐spot boreo‐alpine species can effectively be monitored using noninvasive genetic methods and we highlight potential pitfalls thereof. By detecting a European hare at a high elevation, we emphasize the importance of integrating hybrid identification into future mountain hare monitoring projects, especially in areas with sympatric occurrence of both species. As climate change is expected to increase the ratio of sympatric occurrence, we consider the mountain hare to be an excellent model species for assessing how increasing ambient temperatures and a possible upward shift of a competitive, phylogenetically related lowland species may jeopardize rare high‐elevation species that are pushed toward their upper range limits with restricted habitat availability.

## CONFLICT OF INTERESTS

We have no conflict of interest to declare.

## AUTHOR CONTRIBUTIONS

**Laura Schenker:** Data curation (equal); formal analysis (lead); investigation (lead); software (equal); validation (equal); visualization (lead); writing – original draft (lead); writing – review & editing (lead). **Kurt Bollmann:** Conceptualization (lead); methodology (equal); project administration (lead); resources (equal); supervision (equal); writing – original draft (equal); writing – review & editing (equal). **Maik Rehnus:** Conceptualization (equal); funding acquisition (equal); methodology (equal); resources (equal); supervision (equal); writing – original draft (equal); writing – review & editing (equal). **Sabine Brodbeck:** Data curation (equal); formal analysis (equal); methodology (equal); validation (equal); writing – original draft (supporting); writing – review & editing (supporting). **Felix Gugerli:** Data curation (lead); formal analysis (equal); investigation (equal); methodology (equal); software (equal); supervision (lead); validation (equal); writing – original draft (equal); writing – review & editing (equal).

## Supporting information

Appendix S1‐S2

## Data Availability

The following data are accessible on Dryad (https://doi.org/10.5061/dryad.1vhhmgqqm): Sampling locations (coordinates) of the systematic sampling grid; nDNA microsatellite genotypes (raw genotype data and consensus genotypes of samples with associated individual IDs) from noninvasive sampling; Genotypes of tissue samples with morphologically assigned species identification (*L. timidus, L. europaeus*, putative hybrids); R script used to generate consensus genotypes based on raw genotype replicates; R script used to determine sex of individuals.
