# Supplementary material for: Hare's affairs: Lessons learnt from a noninvasive genetic monitoring for tracking mountain hare individuals
Source: Ecol Evol. 2020 Sep 4;10(18):10150–66. doi: 10.1002/ece3.6676 (PMC7520196; doi:10.1002/ece3.6676)
Supplement: Supplementary file 1 — Appendix S1‐S2 [file ECE3-10-10150-s001.docx]

# Appendix S1


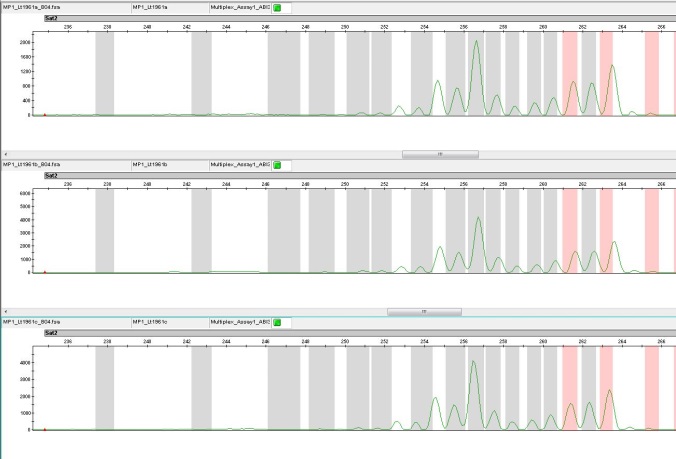

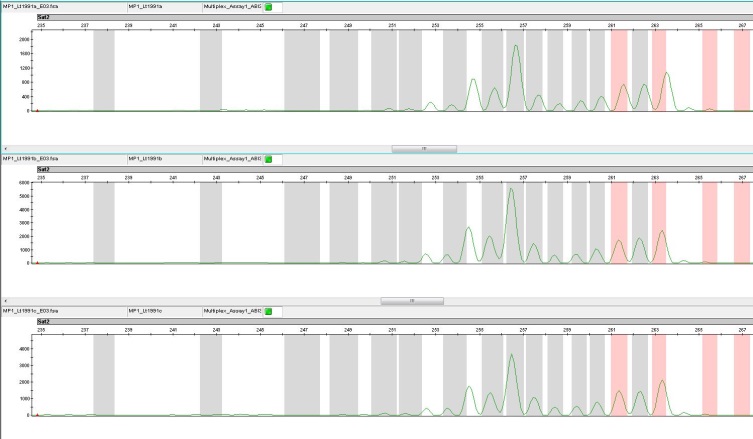


**Figure S1**: Allele peak patterns of mountain hares (*Lepus timidus*) for Sat2 in GeneMapper (Thermo Fisher Scientific, Waltham, Massachusetts, USA) classified as Individual ID57, male. One row gives one replicate, columns give the genotypes of two different samples assigned to the same individual.


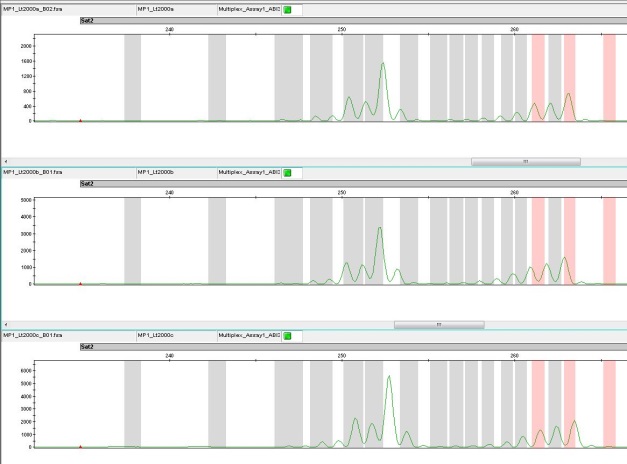

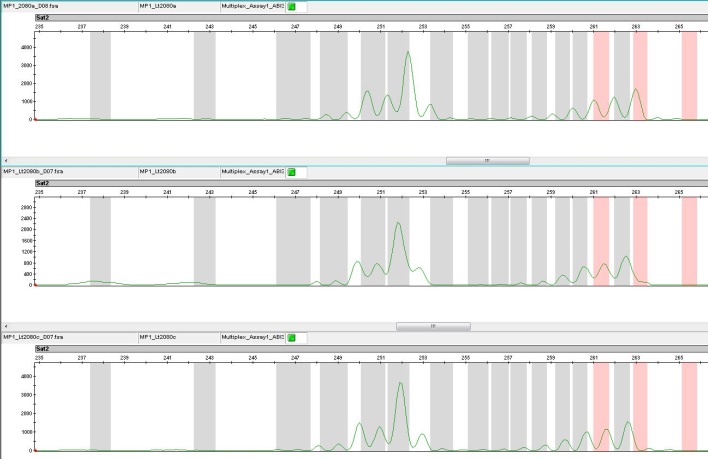


**Figure S2:** Allele peak patterns of mountain hares (*Lepus timidus*) for Sat2 in GeneMapper (Thermo Fisher Scientific, Waltham, Massachusetts, USA) classified as Individual ID59, male. Replicates are given in rows, columns give the genotypes of two different samples assigned to the same individual.


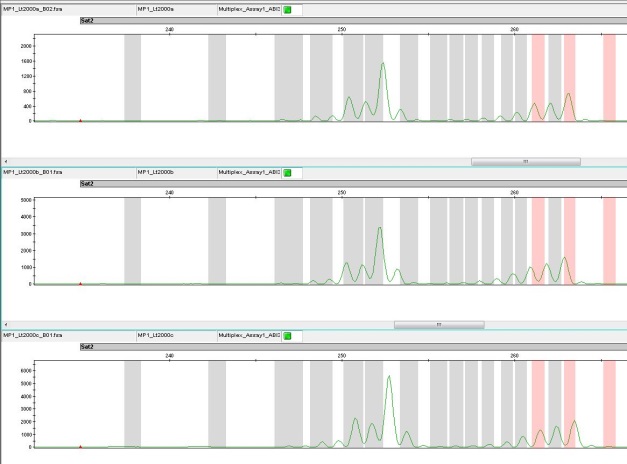

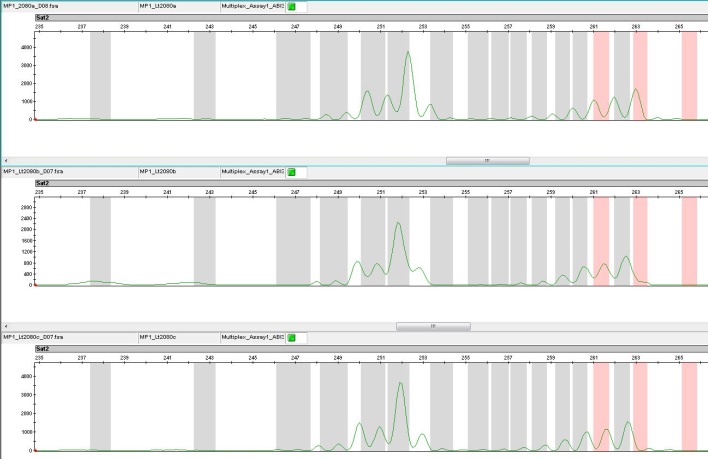
**Figure S3:** Allele peak patterns of mountain hares (*Lepus timidus*) for Sat2 in GeneMapper (Thermo Fisher Scientific, Waltham, Massachusetts, USA) classified as Individual ID15, female. Replicates are given in rows, columns give the genotypes of two different samples assigned to the same individual.


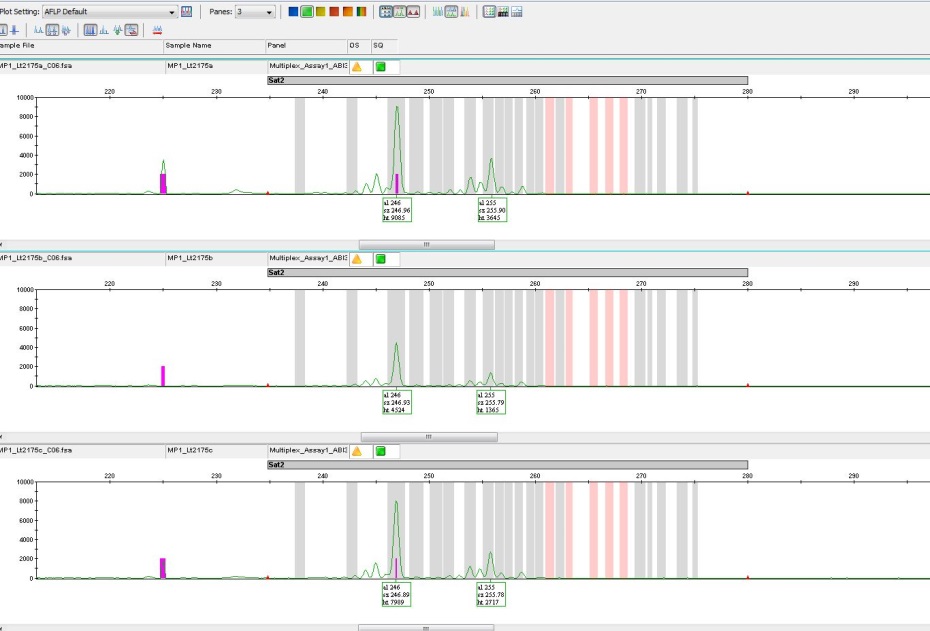

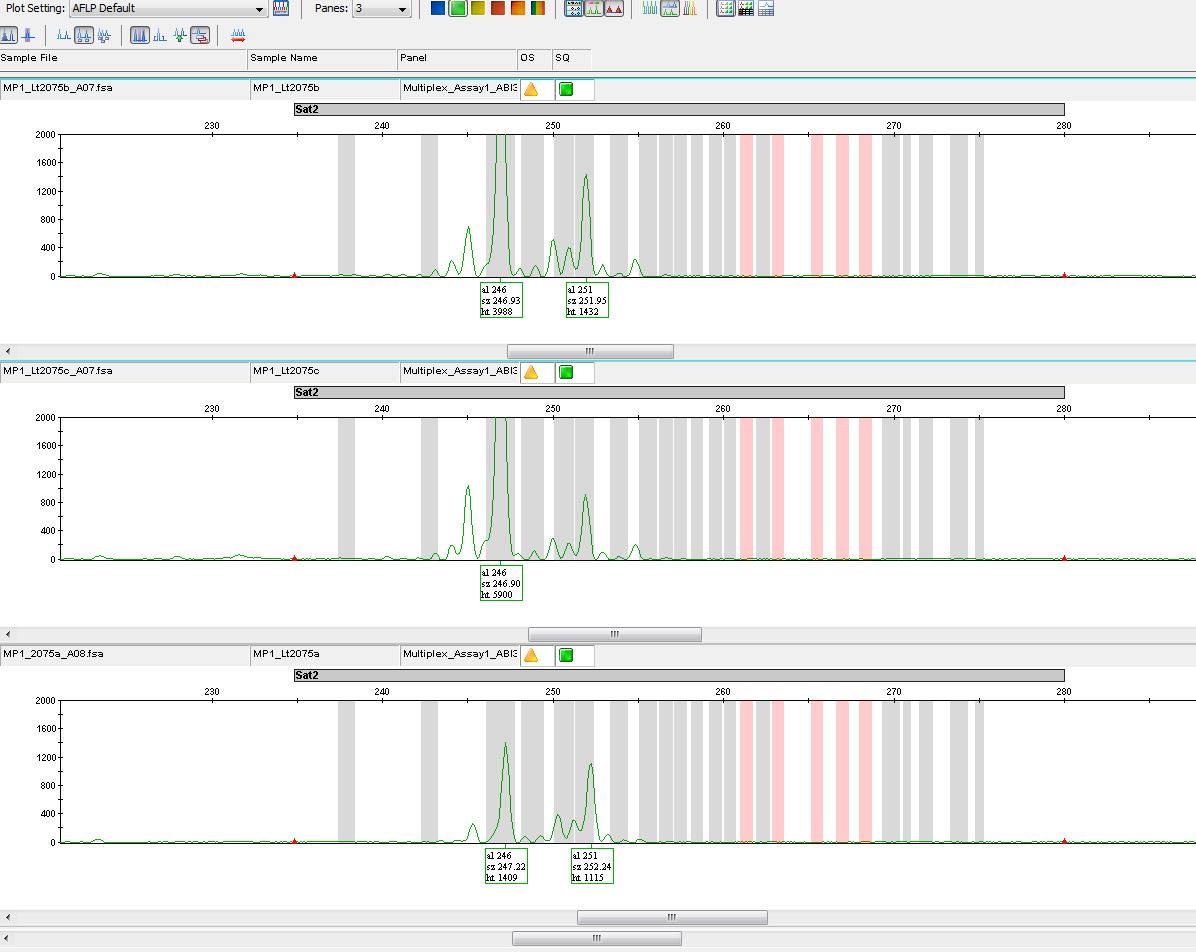


**Figure S4:** Allele peak patterns of mountain hares (*Lepus timidus*) for Sat2 in GeneMapper (Thermo Fisher Scientific, Waltham, Massachusetts, USA) classified as two different individuals based on the peak patterns shown. Each replicate is given in a separate row, columns give the genotypes of two different samples, sample numbers are given in the top left corner.

#
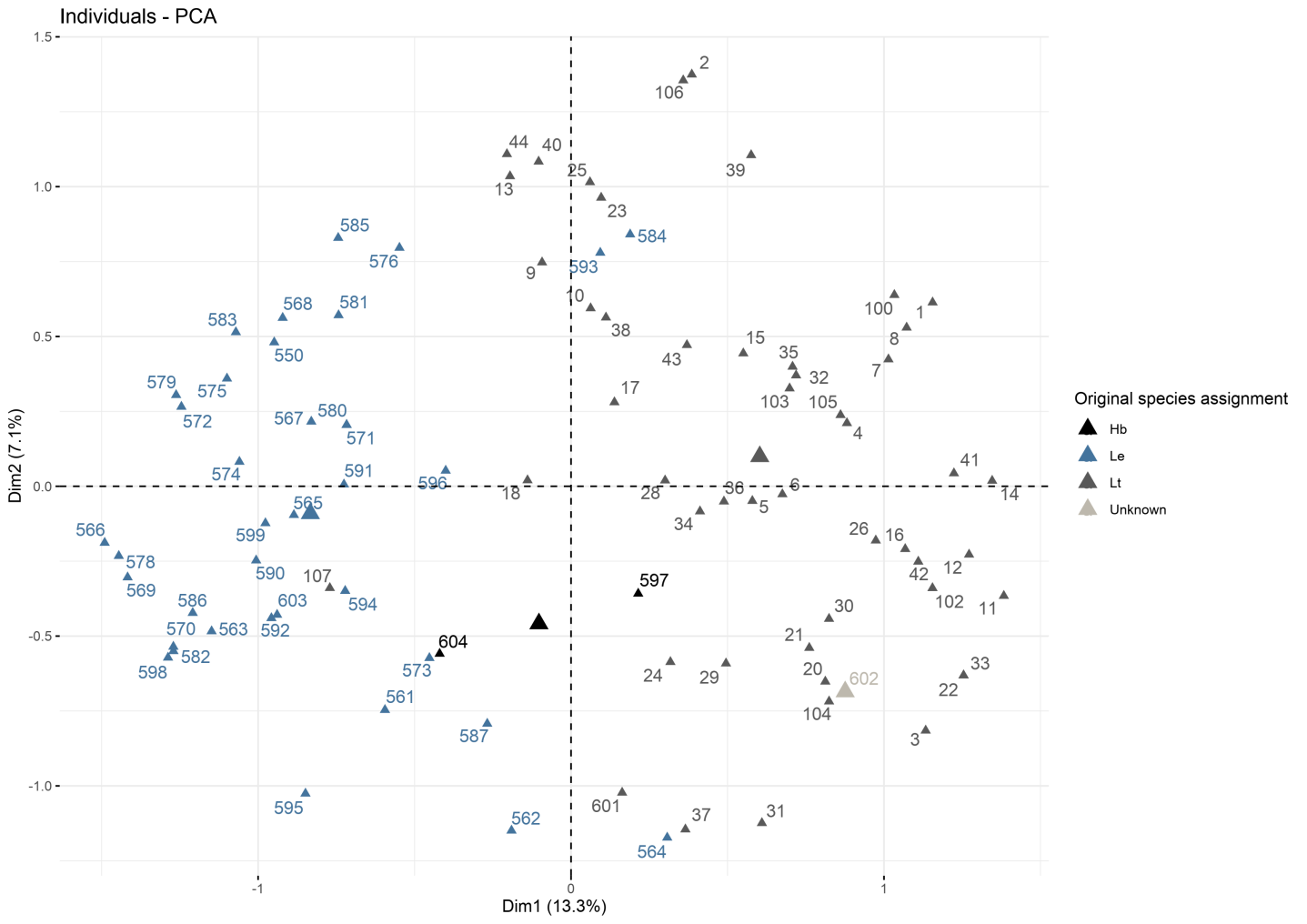
Appendix S2

**Figure S5**: Results of the principle component analysis (PCA) using only tissue samples with morphologically assigned species identities. *Lepus timidus* (Lt) are shown in dark grey, *Lepus europaeus* (Le) in blue, assumed hybrids (Hb) in black and genotypes from samples with an unknown species identity (Unknown) in light grey. Small symbols denote individual samples, while large samples represent the centroid of the respective group of samples.
